# Robotic Stroking on the Face and Forearm: Touch Satiety and Effects on Mechanical Pain

**DOI:** 10.3389/fpain.2021.693987

**Published:** 2021-11-22

**Authors:** Pankaj Taneja, Lene Baad-Hansen, Sumaiya Shaikh, Peter Svensson, Håkan Olausson

**Affiliations:** ^1^Section of Oral and Maxillofacial Surgery, Department of Dentistry and Oral Health, Aarhus University, Aarhus, Denmark; ^2^Scandinavian Center for Orofacial Neurosciences, Aarhus, Denmark; ^3^Section of Orofacial Pain and Jaw Function, Department of Dentistry and Oral Health, Aarhus University, Aarhus, Denmark; ^4^Department of Biomedical and Clinical Sciences (BKV), Center for Social and Affective Neuroscience, Linköping University, Linkoping, Sweden; ^5^Department of Orofacial Pain and Jaw Function, Faculty of Odontology, Malmo University, Malmo, Sweden

**Keywords:** orofacial pain, satiety, pleasantness, unpleasantness, C-tactile afferent

## Abstract

**Background:** Slow stroking touch is generally perceived as pleasant and reduces thermal pain. However, the tactile stimuli applied tend to be short-lasting and typically applied to the forearm. This study aimed to compare the effects of a long-lasting brushing stimulus applied to the facial region and the forearm on pressure pain thresholds (PPTs) taken on the hand. Outcome measurements were touch satiety and concurrent mechanical pain thresholds of the hand.

**Methods:** A total of 24 participants were recruited and randomized to receive continuous stroking, utilizing a robotic stimulator, at C-tactile (CT) favorable (3 cm/s) and non-favorable (30 cm/s) velocities applied to the right face or forearm. Ratings of touch pleasantness and unpleasantness and PPTs from the hypothenar muscle of the right hand were collected at the start of stroking and once per minute for 5 min.

**Results:** A reduction in PPTs (increased pain sensitivity) was observed over time (*P* < 0.001). However, the increase in pain sensitivity was less prominent when the face was stroked compared to the forearm (*P* = 0.001). Continuous stroking resulted in a significant interaction between region and time (*P* = 0.008) on pleasantness ratings, with a decline in ratings observed over time for the forearm, but not on the face. Unpleasantness ratings were generally low.

**Conclusion:** We observed touch satiety for 5 min of continuous robotic brushing on the forearm confirming previous studies. However, we did not observe any touch satiety for brushing the face. Mechanical pain sensitivity, measured in the hand, increased over the 5-min period but less so when paired with brushing on the face than with brushing on the forearm. The differential effects of brushing on the face and forearm on touch satiety and pain modulation may be by the differences in the emotional relevance and neuronal pathways involved.

## Introduction

The ability of pain modulation *via* a variety of presentations of pleasant stimuli has been established (1–3). More recently, it has been shown that a pleasant valence attributed to somatosensory stimuli resulted in a reduction of orofacial muscular pain (4) and heat pain (5), when the stimulus was applied to the same dermatome. Within the aforementioned studies, the pleasant stimulus was one, which activated the C-tactile (CT) afferent system (6).

C-tactile afferents are unmyelinated low-threshold fibers located in the hairy skin of humans (7, 8). They have been found to respond vigorously to a slowly moving, innocuous touch, which is commonly produced in situations of closeness or intimacy (9–11). It is therefore not surprising that the activation of CT afferents was positively correlated with the subject-reported pleasantness scores *via* psychometric analyses, and therefore considered to encode for a pleasant sensation (6).

The animal equivalent of the CT afferent is termed C-low-threshold mechanoreceptive (C-LTMR) afferents. The activation of C-LTMRs in rodents has been shown to provide an analgesic role by inhibiting high-threshold C nociceptive input to the dorsal horn (12). While C-LTMRs may have an analgesic effect *via* other means, in mice the release of the molecule TAFA4, a chemokine-like secreted protein, has been shown to suppress the transmission of nociceptive information in the dorsal horn of the spinal cord (13). Furthermore, the positive expectation that tends to accompany a pleasant stimulus, has been reported to activate the opioid system (14) and contributes to a form of placebo-induced pain reduction (15). Individual differences in attachment style may also contribute to the effects of affective touch on pain modulation (16). For instance, the stimulus ideal for CT activation, when delivered by a romantic partner, can signal positive emotions and social support (17), serving to reassure the receiver before experiencing pain and resulting in lower perceived pain. Of course, this would be influenced by the individual's underlying perception of social relationships (18).

The region most often tested in CT afferent studies is the forearm (19), which may be as a result of the high density of CTs present as suggested from their abundance in microneurography recordings (20). However, CT fibers have also been recorded from the facial area (11). Furthermore, their density relates to the density of vellus hairs, which is particularly high in the facial region (21, 22). Hence, coupled with the specialist function of the orofacial region, in particular with caressing, etc., it may be speculated that the positive emotions elicited may be more prominent, resulting in greater experienced pleasantness, in comparison to the forearm, and therefore a greater reduction in pain. However, this is yet to be investigated.

At present, there is extensive literature on pleasant touch, however, the touch applied is commonly short-lasting, likely as a result of the post-activation depression property of CT afferents (8, 23). Following initial activation of CT afferents, they exhibit fatigue, whereby they have a marked decrease in response to a stimulus, and this effect may last for several minutes; justifying why a substantial amount of CT afferent investigations utilize few strokes with a period of rest termed the interstimulus interval. Although only a limited number of studies have investigated continuous, or, prolonged pleasant stimulation, it has been reported that a decrease in pleasantness occurs over time (24).

The study aimed to assess (*via* psychophysics) if a continuous CT favorable (pleasant) dynamic stimulus applied to the facial region can modulate the pain threshold in a differing region of the body, more than a CT favorable dynamic stimulus applied to the forearm. The hypotheses to be tested were (1) a standardized continuous brush stimulus applied to the face is considered more pleasant than a standardized continuous brush stimulus applied to the forearm. (2) A standardized continuous CT favorable brushing stimulus to the face can modulate the pressure pain threshold (PPT) in a different region of the body. (3) A standardized continuous CT favorable brushing stimulus to the face modulates PPT on the hand more than a continuous CT favorable brush stimulus on the forearm. (4) A standardized continuous CT favorable dynamic brushing stimulus (3 cm/s) can modulate PPT more so than a continuous stimulus preferentially targeting Aβ nerve fibers (stroking at 30 cm/s).

## Materials and Methods

### Participants

A total of 24 participants were recruited from the database of the Center for Social and Affective Neuroscience, Linkoping, Sweden, and The Section of Orofacial Pain and Jaw Function, Aarhus, Denmark, to attend two sessions each. Sample-size calculation estimated a 0.86 effect size for the intraindividual difference between pleasant and control stimuli in experimental orofacial muscular pain (4). Therefore, risks of errors for type I at 5% and type II at 20%, resulted in a total sample size of 20.

Four participants withdrew. One suffered a panic attack during the first session but did not disclose the reasoning behind it, and three did not come back for the second visit without stating any reason. Their data were not used for the analyses. Therefore, the included participants consisted of 14 women (age range 18–35 years) and 6 men (age range 18–25 years), all right-handed. They were compensated 200 SEK per hour of their time with testing lasting approximately 3 h. The exclusion criteria consisted of current orofacial pain or undergoing dental treatment, diabetes, neurological diseases/disorders, current use of analgesic medication, or psychiatric disease. The study was performed under the Helsinki Declaration II and approved by The Scientific Ethics Committees for Central Denmark Region (reference 1-10-72-312-16) and Linköping, Sweden (2018/234-32).

### Pressure Pain Stimuli

Utilizing an electronic algometer (SOMEDIC Algometer, SOMEDIC sales AB, Sweden), that responded linearly to force application between 0 and 10 kg with a probe diameter of 1 cm^2^, the pressure was applied to the hypothenar muscle of the right hand. The participant pressed a button when the pain was first felt and the value recorded was referred to as the PPT. The PPT was calculated as the mean of three series of ascending stimulus intensities, each applied as a slowly increasing ramp of 50 kPa/s (25). The two experimenters had previous experience and were familiar with the technique utilized, to deliver the stimuli in a controlled manner.

### Tactile Stimuli

**Figure 1:**
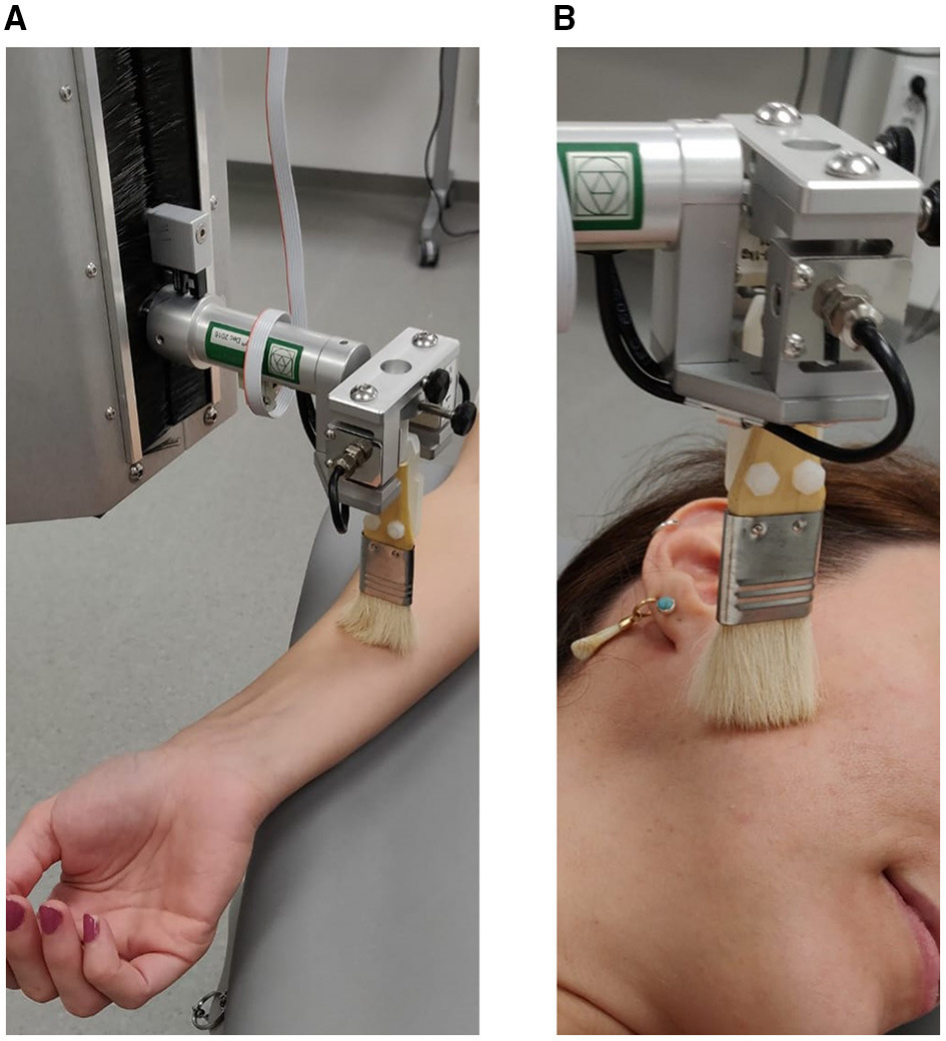
Overview of the experimental setup. Tactile stimulation was delivered by the RTS while the participant was lying flat with right forearm extended **(A)** and head turned to expose right side **(B)**.

A 7-cm-wide goat hair brush was mounted onto a robot, termed the Rotary Tactile Stimulator (RTS, Dancer Design, St Helens, UK). This allowed controlled brush strokes in a pendulum motion to be delivered at a calibrated force of 0.4 N and velocities of 3 and 30 cm/s to the face and forearm (Figure 1). Brush strokes were delivered continuously in a bidirectional rotation (proximal to distal and vice versa).

### Experimental Procedure

Participants were provided with information leaflets before the start of the study. On the day of participation, any questions regarding the study were answered and written consent was obtained.

The study was undertaken in a quiet room without disturbance. First, one PPT measurement trial was performed with the algometer on the hand (the hypothenar muscle of the right hand) to ensure that the measurement procedure was understood. Following that, the region and velocity were randomized, with one velocity and both regions tested per session.

**Figure 2:**
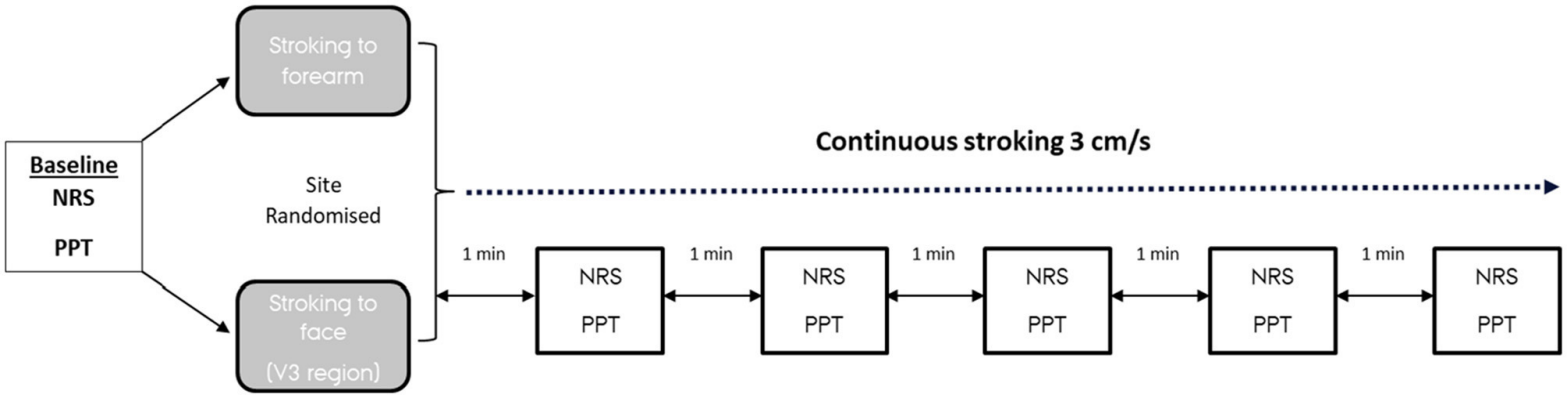
Schematic representation of the experimental design. The velocity randomized would be used for the entire session, in this example 3 cm/s, with 30 cm/s to be used at the second session. NRS, numerical rating scale (0–100 for pleasantness and unpleasantness); PPT, pressure pain threshold (to hypothenar muscle).

Pressure pain thresholds were taken before baseline measurement and at 1 min intervals during the stroking (Figure 2). Initially, a full bidirectional stroke was delivered to the desired region and the participant rated how pleasant [0–100 numeric rating scale (NRS), 0 = no pleasantness of any kind and 100 = the most pleasant sensation imaginable] and unpleasant (0–100 NRS, 0 = no unpleasantness of any kind and 100 = the most unpleasant sensation imaginable) the stroking sensation was perceived (26). The velocity to use in the first session was selected by randomization (either 3 or 30 cm/s). This velocity would then be used on both regions for that session and the other velocity was used in the second session, for each individual. The region, which would first be exposed to the stroking was also randomized. Both randomizations (session and region) were in a balanced design. Ratings were collected at every minute, for a total of 5 min. To avoid the fatigue of the receptors, or potential sensitization, the PPT was measured across three sites of the hypothenar muscle.

The location of stroking on the face was the skin overlying the mandibular branch of the trigeminal nerve ipsilateral to the PPT site. This region was selected for its large area and ease of application of the brush stroke. For the forearm, strokes were delivered to the flattest portion of the dorsal forearm ipsilateral to the PPT site. A break of 30 min was provided to the participant when changing between the different regions to prevent any potential crossover effects.

## Data and Statistical Analyses

All data are presented as mean ± standard error of the mean (SEM).

Model validation was performed by inspecting Q–Q plots.

Pressure pain thresholds, pleasantness, and unpleasantness ratings at the investigated velocities, regions, and times were investigated by a repeated measures three-way ANOVA [velocity (cm/s) × stroking region (face or forearm) × time (min)]. *Post-hoc* analyses were performed by the Tukey's honestly significant difference test with correction for multiple comparisons. A value of *P* < 0.05 was considered statistically significant.

## Results

### Pressure Pain Thresholds

**Figure 3:**
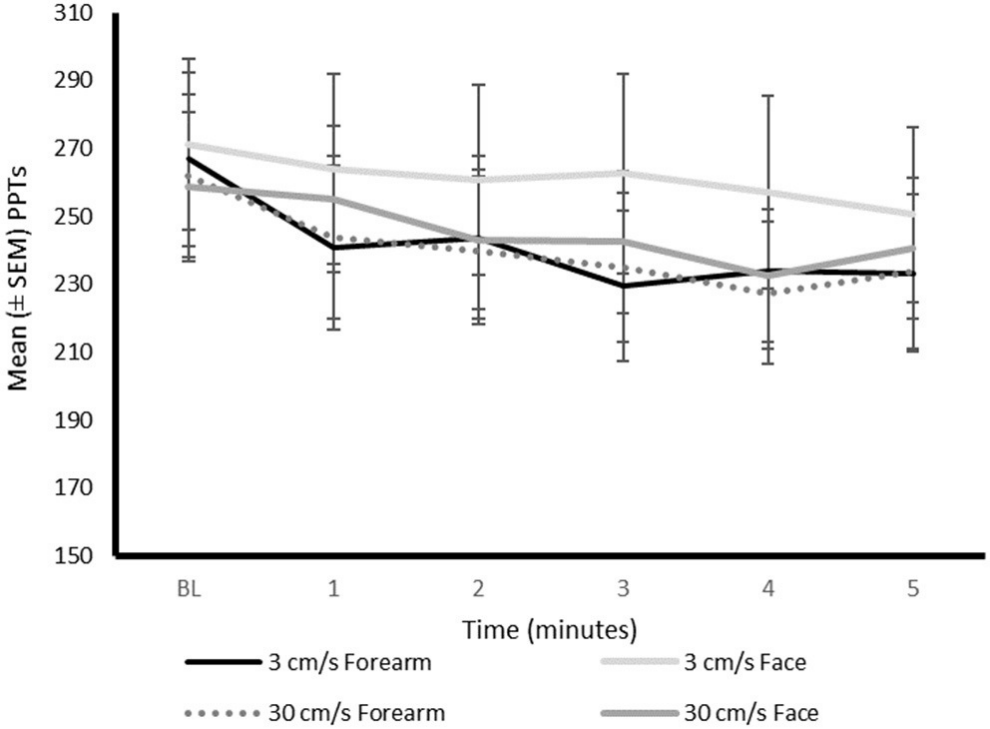
Mean PPTs (kPa) during continuous stroking on the differing regions at the differing time intervals. BL, baseline; NRS, numerical rating scale; PPTs, pressure pain thresholds.

A significant main effect of region (*P* = 0.001) and time (*P* < 0.001) was identified, Figure 3. There was no significant effect of velocity (*P* = 0.310) and no significant interactions between factors (*P* > 0.133).

Overall, PPTs were significantly lower (increased pain sensitivity) when stroking the forearm (mean = 242.91 ± 80.62) compared to the face (mean = 256.02 ± 83.92), irrespective of the stroking velocity and time. An overall decrease in PPT was found over time (*P* < 0.001). The *post-hoc* analysis demonstrated that PPTs were significantly lower at the second, third, fourth, and fifth minute (*P* < 0.008), compared to baseline. Pressure pain thresholds were also significantly lower at minute four compared to the first minute of continuous stroking (*P* < 0.027).

### Pleasantness Ratings

All participants experienced a pleasant sensation when stroked with the brush on both the face and forearm.

**Figure 4:**
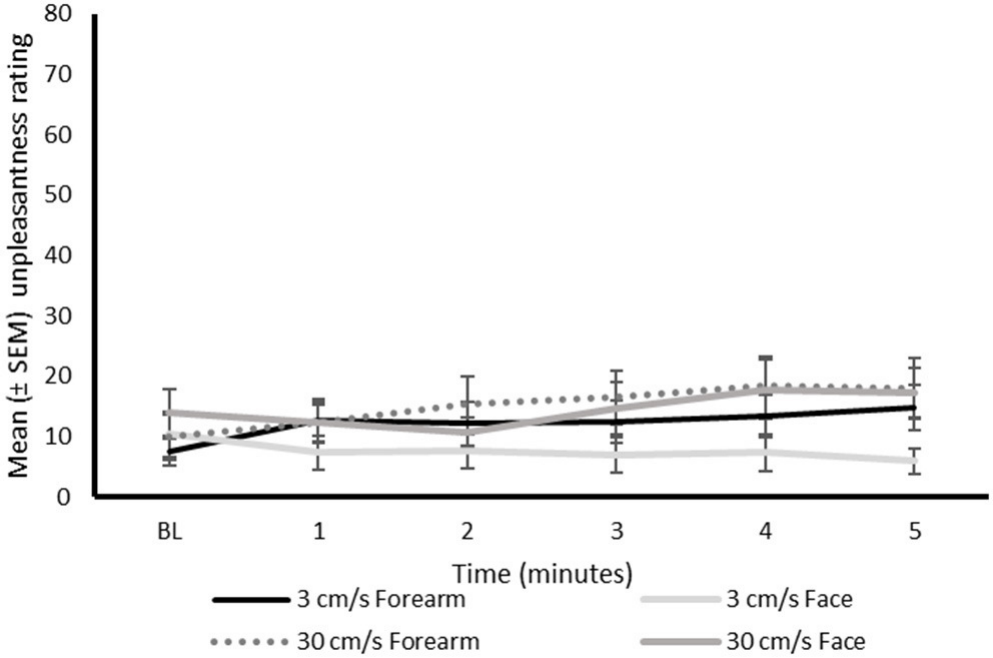
Mean pleasantness ratings (0–100 NRS) during continuous stroking on the differing regions at the differing time intervals. BL, baseline; NRS, numerical rating scale.

A significant main effect of time on mean pleasantness ratings (0–100 NRS) was identified (*P* = 0.046), Figure 4. However, the pair-wise comparisons between time points in the *post-hoc* analysis did not reveal any statistically significant differences (*P* > 0.078). There were no significant main effects of velocity (*P* = 0.118) or region (*P* = 0.904) on pleasantness ratings. A significant interaction between region and time was identified (*P* = 0.008), with *post-hoc* analysis identifying that there was a significant decrease in pleasantness ratings at 3 (*P* = 0.045), 4 (*P* = 0.001), and 5 (*P* < 0.001) min of continuous stroking on the forearm compared to baseline, but not the face (*P* > 0.107). There were significantly lower pleasantness ratings at minutes four (*P* = 0.003) and five (*P* < 0.001), compared to the first minute of continuous stroking of the forearm.

No significant interactions between velocity and region (*P* = 0.614) and velocity and time (*P* = 0.638) were identified.

### Unpleasantness Ratings

**Figure 5:**
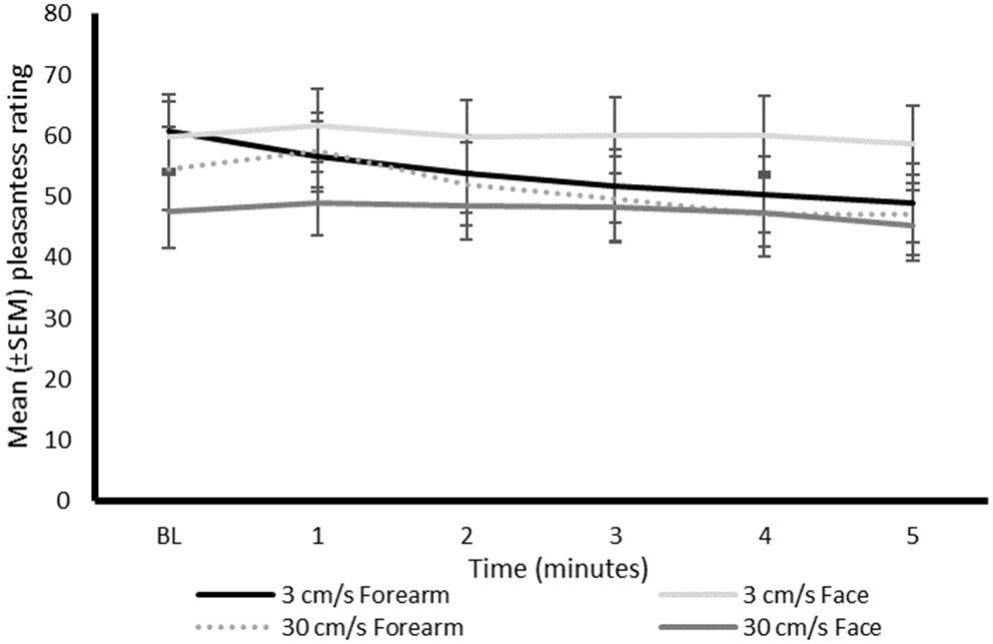
Mean unpleasantness ratings (0–100 NRS) during continuous stroking on the differing regions at the differing time intervals. BL, baseline; NRS, numerical rating scale.

The unpleasantness ratings were generally low (Figure 5). Nevertheless, there was only one participant that did not experience any unpleasantness (i.e., NRS of 0) associated with the continuous stroking at either velocity on the face, and two participants that did not experience unpleasantness at either stroking velocity on the forearm.

There were no significant main effects of velocity (*P* = 0.254), region (*P* = 0.351), or time (*P* = 0.550) on unpleasantness scores (0–100 NRS), Figure 5.

A significant interaction between region and time was identified (*P* = 0.007), with *post-hoc* analysis identifying a significant increase in unpleasantness ratings at 4 (*P* = 0.015) and 5 (*P* = 0.012) min of continuous stroking on the forearm, but not the face (*P* > 0.056), compared to baseline.

No significant interactions between velocity and region (*P* = 0.470) and velocity and time (*P* = 0.379) were identified.

## Discussion

In general, PPTs declined (increased pain sensitivity) over time during continuous stroking, compared to baseline, and there were no differences in the PPTs when continuous stroking was applied at different velocities. However, PPTs on the hand were significantly less reduced when continuous stroking was applied to the face, compared to the forearm. There was no difference in pleasantness ratings between the face and forearm, however, continuous stroking resulted in a modest decline in pleasantness ratings over time on the forearm but not on the face.

It has been identified that a CT favorable stimulus can reduce experimental pain (27). Here, neither CT favorable (3 cm/s) nor Aβ favorable (30 cm/s) stimulation resulted in a decrease in pain sensitivity. However, since we did not have a condition without touch in our experimental design it is possible that both stimuli mitigated pain sensitization. Specific to Aβ fibers, modulating pain by touch stimuli has been attributed to activation of wide dynamic range (WDR) neurons in the dorsal horn of the spinal cord (28). The WDR neurons respond to both nociceptive and tactile inputs, with receptive fields containing excitatory centers and inhibitory peripheries. Within humans, it has been found that the spatial position of the touch stimulus (activating Aβ fibers) can influence the pain relief experienced (29). As pain measurements were taken from the hand, and stimulation was performed on the forearm and face, the distance from the WDR neuron fields may explain the lack of pain reduction between sites. Furthermore, tactile inputs tend to inhibit pain perception segmentally but not when applied to different dermatomes (29), as undertaken in the present study and further supporting the lack of pain-reducing effects observed.

C-tactile favorable touch provides a non-verbal method of communicating social support and results in reduced pain sensitivity (18). In this study, CT-targeted and Aβ-targeted strokes were rated similarly. The hedonic tone, i.e., valence, modulate pain which may explain a similar effect for both CT- and Aβ-targeted touch in this study (4). However, the absence of a social context in the delivered stimulation may account for the absence of pain reduction between the velocities (17). It could be considered that the environment created differs when the stimulus lacks the characteristics that accompany human touch, a factor previously inferred (5, 18).

A prolonged CT specific (24, 30) or non-specific (30) stimulus result in a moderate decline in pleasantness ratings, over time. However, in these studies testing was exclusively performed on the forearm. This study reinforces these findings but adds knowledge from the understudied facial region, suggesting that the site may determine how a continuous stroking stimulus is perceived over time.

The effect of time played a role in both pleasantness and unpleasantness ratings when applied to the forearm. Continuous stroking resulted in a significant decrease in pleasantness ratings by the third minute and a significant increase in unpleasantness ratings by the fourth minute. These changes were not identified when the face was stroked. It could be speculated that the emotional significance of the stroked region may determine the change in perceptions. For example, the face could be considered a highly emotional area and possibly more tolerant of a continuous touch than a differing region, such as the forearm. Nonetheless, the absence of social context in this study may contribute to the findings in the stimulus delivery between sites.

The decrease in reward from repeated exposure, termed satiety, has specifically been investigated for affective touch, specifically on the forearm (31, 32). The affective value of touch was found to decrease over time with an increase in unpleasantness perception (31, 32). Such touch satiety was suggested to be modulated by peripheral and cognitive factors, e.g., boredom and/or the mechanisms linked to the relationship between the subject and experimenter. When exactly the shift in the perception paradigm occurs is still unclear. One factor that may contribute to the uncertainty is the common use of a single visual analog scale which encompasses differing perceptions, i.e., having endpoints that range from unpleasantness to pleasantness (24, 30) (or vice versa). A suggestion for future studies would be to utilize separate scales for each perception as this study has shown, stimuli can encompass a variety of perceptions at the same time.

It has recently been stated that the link between pleasantness and CT afferent activation is still a hypothesized effect (33). The current study indicates that CT afferent activation does not only give rise to sensations of pleasantness but also other emotional attributes which are not necessarily positive. Certainly, it has previously been demonstrated that a CT favorable stimulus has a multidimensional construct that includes both pleasant and unpleasant sensations (4, 26, 34, 35). This aspect may have been overlooked in previous studies investigating CT activation and using single rating scales with endpoints of pleasantness to unpleasantness (or vice versa). To the knowledge of the authors, this has been demonstrated for the first time from a continuously delivered stimulus in this study. It can only be hypothesized that the dominating sensation may depend on the context within which the stimulus is being delivered and/or the phenotype of the individual (33).

It must not be overlooked that both cutaneous and subcutaneous regions will contribute to the recorded changes in PPTs (36). The reduction in PPTs observed from 2 min onward may have resulted from the repetitive measurements, and thereby repeated pressure, of the PPTs on a small muscle causing sensitization of the overlying cutaneous and/or deeper located nociceptors. To exclude part of this effect, and for consideration in future studies, a control group could have been utilized with a topical local anesthetic applied to the overlying skin (37). It is interesting to note that the PPTs decreased significantly less when stroking was applied to the face than when stroking was applied to the forearm. As the continuous stimulation was rated as no different in terms of pleasantness between the sites, and from the lack of social context, any modulatory effect may more likely be attributed to the difference in mechanistic processes, i.e., trigeminal vs. spinal mechanism, as has been reported in other studies indicating heterotopic site differences for pain thresholds (38–40) and the magnitude of endogenous pain inhibition (41). In addition, differences in the density of tactile afferents that innervate the skin may vary between the face and forearm which may, in turn, contribute to the modulation in observed PPTs (42–44).

## Limitations

This study was associated with some limitations.

The RTS is a large robotic device that emits noise when operating. This may have served to distract the participant from the PPT, particularly when stroking was performed on the face and, as a result, the RTS located closer to the ear and generating a greater noise. However, we have previously demonstrated that stroking by the RTS or by a human experimenter gives similar pleasantness ratings (45). In addition, although the force and velocity were controlled by the RTS, calibration was performed on the position of the participants at the start of the experiment. Any movements by the subject during the experiment may have compromised those factors.

Due to time constraints, we were not able to recruit an equal number of male and female participants, and investigation of gender differences was therefore not possible. Since there is a known difference in pain sensitivity between genders (46, 47), it seems possible that there also is a gender difference in pain modulation *via* pleasant touch. Regrettably, this could not be addressed in this study.

Furthermore, the small sample size and narrow age range limit the generalizability, particularly to the elderly population. The PPT measurements were always coupled to a tactile stimulation on either the forearm or the face. Hence, we do not know how the PPTs may have developed over time in the absence of the tactile stimulation, and therefore we do not know to what extent the tactile stimulation reduced pressure pain sensitivity. These factors should all be considered in future studies.

## Conclusion

The threshold to mechanical pain reduced over time, potentially occurring as a result of sensitization. However, this sensitization was less prominent when the face was stroked compared to the forearm and may attribute to the emotional relevance of the facial region, or the processing of information *via* the trigeminal pathways.

The pleasantness experienced from a continuous stroking stimulus declined over time only on the forearm and not on the face, suggesting that touch satiety may be region-specific and potentially dependent on the emotional relevance and neuronal pathways (i.e., trigeminal or spinal) involved.

## Data Availability Statement

The raw data supporting the conclusions of this article will be made available by the authors, without undue reservation.

## Ethics Statement

The studies involving human participants were reviewed and approved by the Scientific Ethics Committees for Linköping, Sweden (2018/234-32) and the Scientific Ethics Committees for Central Denmark Region (reference 1-10-72-312-16). The patients/participants provided their written informed consent to participate in this study.

## Author Contributions

PT, SS, LB-H, and HO contributed to the design of the study. PT undertook data collection. PT, SS, LB-H, PS, and HO contributed to data analysis. All authors contributed to drafting of the manuscript. All authors have read and approved the manuscript.

## Funding

This study was supported by the Danish Dental Association, Aarhus University Research Foundation, and Swedish Research Council. All funding agencies provided funding for participant compensation, payment for experimenters and costs toward use of equipment and facilities.

## Conflict of Interest

The authors declare that the research was conducted in the absence of any commercial or financial relationships that could be construed as a potential conflict of interest.

## Publisher's Note

All claims expressed in this article are solely those of the authors and do not necessarily represent those of their affiliated organizations, or those of the publisher, the editors and the reviewers. Any product that may be evaluated in this article, or claim that may be made by its manufacturer, is not guaranteed or endorsed by the publisher.
